# Aplastic Anaemia with Post-Transfusional Haemosiderosis

**Published:** 1959-04

**Authors:** T. F. Hewer


### APLASTIC ANAEMIA WITH POST-TRANSFUSIONAL HAEMOSIDEROSIS

*A Clinical Pathological Conference of the University of Bristol Medical School*

CHAIRMAN: PROFESSOR T. F. HEWER

*Professor Hewer:* I will ask Dr. Macrae, who first looked after this patient, to give us the clinical history.

*Dr. J. Macrae:* This lady, aged 63, had no previous hospital admission or serious illness. Two months before admission to Ham Green Hospital she had had thrombosis in her left leg and recovered satisfactorily after bed rest. Three weeks before admission she complained of breathlessness on exertion, her heart beat was irregular, her doctor gave her digitalis and she improved. Some three days before admission she complained of what she called "a cold" with cough. She had pain in the right side of the chest and she coughed up blood stained sputum.

She was admitted on 27th July 1957 and was found to have a temperature of 99·2°F; respirations 30 per minute; pulse 88, highly irregular and fibrillating. There was a faint yellowish tinge to the skin but no definite jaundice. Blood pressure was 115/80. No clinical enlargement of the heart was noted but a small right pleural effusion was present. The sputum was flecked with blood, the right leg was rather discoloured, no oedema was present, and the clinical picture suggested that she had had a pulmonary embolism causing pulmonary infarction on the right side.

The chest was X-rayed and the effusion confirmed with some enlargement of the heart noted in addition, and it seemed possible that there were some patches of pneumonia at the left base. The patient was very constipated and she felt much better after a highly successful enema. She was treated with rest, penicillin and digitalis.

Laboratory investigation showed the haemoglobin to be 77 per cent and the pleural fluid, which was brownish in colour, contained no carcinoma cells. She appeared to improve but on the 2nd August she complained again of severe right-sided pain and developed a pleural rub at the left base and we rather assumed that she had had another, although smaller, pulmonary infarction. Thereafter her pulmonary condition improved steadily and did not enter into the clinical picture again.

However, during the same time it was noted that haemoglobin percentages were going down steadily and, by the middle of September, the haemoglobin was 53 per cent. She had been receiving ferrous sulphate orally since the end of August, but, as this did not seem to improve the haemoglobin, she received "Imferron" 5 c.c. twice weekly from 10th September onwards to a total of eight doses. The blood picture on 18th September showed: haemoglobin 52 per cent; red blood cells 2,500,000; white blood count 5,200; polymorphs 68 per cent; lymphocytes 22 per cent; mononuclears 7 per cent; eosinophils 3 per cent.

Sternal marrow was obtained on 20th September and Dr. Bolton reported: "This is a moderately cellular marrow. It is normoblastic and shows no evidence of iron deficiency or of megaloblastic haemopoiesis. The peripheral blood film shows increased red cell regeneration".

It was suggested that the patient was suffering from concealed haemorrhage of some sort, but we could find no site of haemorrhage; in particular occult blood was never found in the stools, although tested many times. Serum bilirubin was 1·4 mgms. per cent; blood urea 33·3 mgms. per cent, and on 23rd September 1958 reticulocytes were 3·7 per cent and the haemoglobin was 53 per cent.

On 2nd October 1957 the patient was transfused with one and a half pints of packed cells. Shortly after transfusion she developed some small patches of purpura on the chest, buttocks and legs. The haemoglobin on 9th October 1957 was 70 per cent with red blood cells 3,010,000; white blood count 6,000; platelets 30,100. Coombs's test was negative.

The patient felt much better; she had got tired of being in hospital and wanted t go home. Although we had still found no reason for her anaemia, we allowed her to g and I told her general practitioner that she would probably need further investigatio and we would have her back.

On 30th October 1957 she was re-admitted, pale, breathless with a haemoglobi of 38 per cent; platelets 41,000 per cu. mm. She was given a transfusion of one pi packed cells and transferred to the care of Dr. Cates at the Bristol Royal Infirmary o 6th November 1957 as a case of probable aplastic anaemia.

*Dr. J. E. Cates:* When she came in we had the full history from Dr. Macrae, w checked on one or two points, and turned to the patient. We found that she was indee very pale, fat and poorly. She was short of breath even when turning over in be Though she was apathetic, she retained her sense of humour. She was covered wit purpura over her shoulders and chest. Temperature was between 99 and 100° Her pulse and respiration were normal. There was bruising on her arm where bloo pressure had been measured. There was no jaundice, a feature you might find i haemolytic anaemia. There were no enlarged lymph nodes, and no enlarged splee to fit in with a diagnosis of leukaemia. Her heart was slightly enlarged with a raise venous pressure, some peripheral oedema and rales at the bases. Her haemoglobi was 42 per cent, E.S.R. 69 mm/hour. Total white cell count 1,700 (polymorphs 60 and reticulocytes below 0·6; platelets 10,000/cu. mm. and a normochromic film. Th stools contained occult blood; we interpreted this as meaning that she had som scattered oozing of blood from her bowels. All this seemed to fit in with Dr. Macrae suggestion that her bone marrow was failing. White cells and platelets were reduce suggesting that she had panmyelophthisis. There was no bleeding to cause anaemia an no evidence of haemolysis.

A further bone marrow biopsy was done by Dr. Bolton but the report was no conclusive.

We then tried to find out why she should have developed marrow aplasia. W telephoned Dr. Macrae and asked what her treatment had been. We were intereste to know if the patient had had chloramphenicol or sulphonamides for her pneumon or if she had had any chlorpromazine.

An important danger to bear in mind is benzene, for there is always the possibili of washing paint or clothes in it; we found no story of any toxic substance. We wo dered if this marrow aplasia could be due to something else, for example secondary uraemia. She did not look uraemic clinically. There was no acidosis and the laborato findings excluded this possibility. We wondered about D.L.E. because she had a hi sedimentation rate but we found that she had no other clinical evidence to suppo this diagnosis. Thus her heart was normal, her urine was normal, and there was arthritis. Plasma proteins were not much abnormal and the L.E. test was negative several occasions.

We transfused her with blood at the rate of 4 pints in the first three days; we had watch her very carefully as she was in heart failure and rapid transfusion is likely kill people if they have heart failure. We also gave her mersalyl and kept her propp up until she had a high haemoglobin.

In the next 4 weeks in hospital she had a total of 8 pints and was obviously very mu improved by it. She developed a chest infection at both bases so she was given a cou of antibiotics and improved. She then had a tender right calf and pain in the ch and we thought this was a pulmonary embolism. She stopped fibrillating soon af admission, but this began again. We gave her two more pints and she then went ho for Christmas; she was admitted again shortly afterwards and in January she had t more pints and in February we stopped transfusing her.

In February before she actually died she became very drowsy and on 17th Februa had two fits and her respiration rate became raised. It was noticed that she had a ri upper motor facial nerve weakness at that time. Next day she had some more fits a spasmodic twitching of the right arm. On the 19th she developed right-sided he plegia; both toes went up but it was more definite on the right; she then had a series of fits and died in coma. We presumed she had some form of haemorrhage into the brain.

Summing up, this was an old lady of 63 who had a chest infection and developed a progressive anaemia after this with haemorrhages and low platelet count, low reticulocyte count and a hypoplastic marrow. We thought that this aplasia was "ideopathic". Survival was prolonged by repeated blood transfusion and antibiotics. We noticed towards the end that besides being anaemic she was rather earthy in colour, but we did not investigate this to see whether the blood we had given her was doing anything odd.

*Professor Hewer:* You say that there were no drugs given at all which might have had a bearing on the aplasia. In the notes it said that 15 grams of sulphatriad were administered at Ham Green.

*Dr. Cates:* I cannot imagine Dr. Macrae giving her sulphatriad.

*Dr. Macrae:* No, we didn't.

*Dr. M. E. H. Halford:* Was there any evidence that she had destroyed the transfused blood?

*Dr. Cates:* We thought the rate of destruction when transfusion stopped was about as fast as one would expect it normally to be. There did not seem to be any haemolysis. People having blood transfusions do tend to destroy the older cells in the bottle rapidly. Her haemoglobin fell to 35 from 80 per cent in about 5 weeks, about the rate one would expect with somebody not making any of her own. The mean survival of transfused red cells is 60 days, if given fresh.

*Dr. O. C. Lloyd:* Can you give us some idea about how often you meet such a case and how often do you have to admit that it is idiopathic?

*Dr. Cates:* In about half the cases.

*Dr. G. H. Tovey:* Yes, in about 50 per cent.

*Professor Hewer* describing the post mortem: This woman had a great many petechiae and bruises. The cause of death was easy to determine, it had already been envisaged clinically. There was a massive subdural haemorrhage on both sides, slightly more on the left than the right. There was also subarachnoid haemorrhage. The haemorrhage probably came from superficial veins. There was haemorrhage around the base of the brain and it was considerably compressed by haemorrhage. I think this caused the coma. There were no haemorrhages inside the brain on section. There were no vascular lesions. The whole of the neurological findings and the cause of death could be attributed to the haemorrhage.

The bone marrow, as you would have expected, was extremely pale: there was no red marrow in femur, sternum, ribs or vertebrae. Histologically a few cellular areas were found but these consisted only of groups of lymphocytes and plasma cells. This fits in with the haematological findings during life.

The right pleural cavity was entirely obliterated by old fibrous adhesions, with a solid mass of scar tissue just over the diaphragm and another, with some brown patches of haemosiderin, over the lower lobe posteriorly. In this lower lobe was a pulmonary infarct that was becoming absorbed and there were several patches of bronchopneumonia. Near the hilum of the lung was a partially organized thrombus attached to the wall of a pulmonary artery. This was a nice confirmation of Dr. Macrae's diagnosis of pulmonary embolism. There was also an extensive terminal bronchopneumonia.

The liver had some cough grooves on its surface and since Dr. Macrae says she had no cough until she was admitted to hospital it is interesting to observe that these grooves could be produced within seven months. The liver also had a rusty colour as a result of the presence of a great deal of haemosiderin in the portal zones of the liver and in the Kupffer cells. There was no scarring. Iron was also present in the spleen, pancreas, thyroid and pituitary and in many other situations: nowhere had it provoked any fibrosis.

There were many submucous haemorrhages in the stomach but none in the intestines.

I think one can recapitulate the course of events to some extent. She originally had some venous thrombosis in her legs and this caused several pulmonary emboli. They were probably associated with some low grade pulmonary infection. She then developed an aplastic anaemia as a result of total aplasia of the bone marrow. The cause of this marrow aplasia is unknown, but it may have been due to some drug she obtained from a chemist: we have no evidence. The blood transfusions and iron injections accounted for the haemosiderosis. The absence of megakaryocytes in the bone marrow probably accounted for the fall in blood platelets and her numerous haemorrhages helped to dispose of some of the blood that she was receiving. Eventually spontaneous intra-cranial haemorrhages caused death.

*Dr. Cates:* To go back to sulphatriad, I think we rang up the family doctor and asked how the bad cold had been treated, and I think that is where it came in.

*Dr. Coles:* Novalgin, which contains amidopyrine, is an antipyretic which is, I believe, available at some grocers. Did she have this?

*Professor Hewer:* That is a very nice suggestion of Dr. Coles's—that she took the drug to relieve the pain caused by venous thrombosis and the drug caused her anaemia. Unfortunately we have no evidence that she took any such drug.

*Dr. Cates:* That is so: we obtained no evidence that she had in fact had any amidopyrine.

